# Computational identification of protein-protein interactions in model plant proteomes

**DOI:** 10.1038/s41598-019-45072-8

**Published:** 2019-06-19

**Authors:** Ziyun Ding, Daisuke Kihara

**Affiliations:** 1 Department of Biological Sciences, Purdue University, West Lafayette, IN 47907 USA; 2 Department of Computer Science, Purdue University, West Lafayette, IN 47907 USA; 3 Department of Pediatrics, University of Cincinnati, Cincinnati, OH 45229 USA

**Keywords:** Machine learning, Proteome informatics

## Abstract

Protein-protein interactions (PPIs) play essential roles in many biological processes. A PPI network provides crucial information on how biological pathways are structured and coordinated from individual protein functions. In the past two decades, large-scale PPI networks of a handful of organisms were determined by experimental techniques. However, these experimental methods are time-consuming, expensive, and are not easy to perform on new target organisms. Large-scale PPI data is particularly sparse in plant organisms. Here, we developed a computational approach for detecting PPIs trained and tested on known PPIs of *Arabidopsis thaliana* and applied to three plants, *Arabidopsis thaliana*, *Glycine max* (soybean), and *Zea mays* (maize) to discover new PPIs on a genome-scale. Our method considers a variety of features including protein sequences, gene co-expression, functional association, and phylogenetic profiles. This is the first work where a PPI prediction method was developed for is the first PPI prediction method applied on benchmark datasets of *Arabidopsis*. The method showed a high prediction accuracy of over 90% and very high precision of close to 1.0. We predicted 50,220 PPIs in *Arabidopsis thaliana*, 13,175,414 PPIs in corn, and 13,527,834 PPIs in soybean. Newly predicted PPIs were classified into three confidence levels according to the availability of existing supporting evidence and discussed. Predicted PPIs in the three plant genomes are made available for future reference.

## Introduction

Identification of protein-protein interactions (PPIs) is important for understanding how proteins work together in a coordinated fashion in a cell to perform cellular functions. PPIs are essential for individual protein functions, forming various cellular pathways, and are also involved in the development of diseases. PPI data is directly useful for identifying protein multimeric complexes^1,2^, identifying biological pathways as well as predicting protein function^3–5^. For more on the application side, PPIs are also important targets for drug design^6^ and artificial design of protein complexes^7^.

There are experimental methods for determining individual PPIs, such as co-immunoprecipitation^8^, fluorescence resonance energy transfer^9^, and surface plasmon resonance^10^. Ultimately, biophysical methods such as nuclear magnetic resonance spectroscopy (NMR)^11,12^, X-ray crystallography^13^, and electron microscopy^14^, can be used to determine the tertiary structure of protein complexes to obtain detailed atomic or molecular level information about how the proteins interact. Moreover, from late 1990’s, PPIs have been determined in a large-scale using yeast-two hybrids^15–18^ and affinity chromatography combined with mass spectrometry^19–22^. However, experimental methods have several shortcomings for detecting PPIs. First, these experimental methods are time-consuming and labor-intensive. Second, the applicability of experimental methods depends on how well assay protocols are established in target organisms. Also, a method may not work on some classes of proteins^23,24^. Third, it is known that experimental methods often have difficulty in identifying weak interactions, which leaves out many transient interactions^25^. Fourth, it is discussed that the results of large-scale methods often have a substantial disagreement with each other, which may be partly due to false positives and false negatives^26–28^.

Consequently, PPIs have been identified only for a limited number of organisms; moreover, the coverage of PPI networks is very small for the majority of organisms. This is particularly true for plant species. Table 1 shows the statistics of experimentally identified PPIs in representative plant species taken from the BioGRID database^29^. Surprisingly, except for *Arabidopsis thaliana*, virtually no other plant species have experimentally determined PPI data available. Even for *Arabidopsis*, known PPI data cover interactions with only about 34.55% of proteins. Other representative plant species cover even less protein involved in known PPIs. Thus, it is apparent that plants are largely lagged behind from PPI studies. In this omics era when various types of large-scale data are combined and used for formulating hypotheses and to interpret experimental data, PPI networks are fundamental reference data to have for studying an organism.

**Table 1:** Statistics of the number of experimentally determined PPIs in representative plant species.

Organism	Common Name	Number of Protein genes	Identified unique PPIs	Unique Proteins in PPIs	Fraction of proteins involved in known PPIs (%)
*Arabidopsis thaliana*	mousear cress	27,636	35,908	9,574	34.55
*Zea mays*	corn	37,376	13	21	0.056
*Glycine max*	soybean	46,993	39	43	0.092
*Oryza sativa* (Japonica)	rice	35,679	90	72	0.202
*Solanum lycopersicum*	tomato	25,613	107	44	0.172
*Solanum tuberosum*	potato	28,463	2	3	0.011

The statistics of PPIs were taken from the BioGRID database. The numbers of protein genes were taken from the KEGG database.

To complement experimental methods for identifying PPIs, several computational methods have been developed^30^. These methods typically use a machine learning framework and consider various features of proteins as input. Protein features used for PPI prediction include occurrence of functional domains^31–33^, short sequence patterns (e.g. n-grams, auto-covariation)^34–38^, interlog (interaction inferred from homology)^39–43^, codon usage^44^, function^45,46^, similarity in phylogenetic trees^47–49^, phylogenetic profiles^50^, gene expression^51^, and protein tertiary structures^52–57^. Although many approaches were explored, there are not many works that applied developed methods to provide new proteomics-scale PPI predictions. Existing works are mainly limited to microbial genomes and eukaryotes^40,43,56,58–65^, and only applications to the plant domain are for *Arabidopsis thaliana*^61,63,64^ and *Oryza sativa* (rice)^43,63^.

In this work, we developed a computational method for PPI prediction, named PPIP (PPI prediction for Plant genomes) and applied to three major plant proteomes, *Arabidopsis thaliana*, *Zea mays* (corn), and *Glycine max* (soybean). To capture different aspects of proteins that are relevant to PPIs, we used a combination of four features for predicting PPIs, i.e. protein sequence properties, protein functional similarity, co-expression patterns, and phylogenetic profile similarity. To provide a confidence level of predictions, two machine learning methods, support vector machine (SVM) and random forest (RF), were separately trained on different features, and commonly predicted PPIs by the two methods were considered to have high confidence. The machine learning methods were trained on known PPIs from *Arabidopsis*. The accuracy on the testing dataset of *Arabidopsis* achieved a high accuracy of over 90%. PPIP predicted 50,220, 13,175,414, and 13,527,834 confident PPIs in *Arabidopsis*, corn, and soybean, respectively.

Examples of predicted novel PPIs with high confidence are discussed. All confident predictions are provided on our lab website (http://kiharalab.org/PPIP_results/) so that they can be referenced by plant biologists.

## Results

### Constructing a benchmark dataset of known *Arabidopsis* PPIs

First, we tested two machine learning prediction algorithms in our prediction method, PPIP, namely, support vector machine (SVM) and random forest (RF) on the dataset of known *Arabidopsis* PPIs obtained from the TAIR database^66^ (Additional File 1: Table S1). These PPIs were determined by experiments including X-ray crystallography, affinity-capture mass spectrometry, co-immunoprecipitation, fluorescent resonance energy transfer, isothermal titration calorimetry, and surface plasmon resonance. The downloaded known *Arabidopsis* PPI dataset contained 4,908 PPIs, which were reduced to 4,759 PPIs after removal of short proteins of less than 50 amino acid residues and PPI identified by genetic experimental systems.

To train and test a machine learning method, we also need a negative dataset, i.e. a dataset of protein pairs that do not interact. We construct negative sets in two different ways as mentioned by Guo^67^. One is to pair proteins from different cellular localizations and thus highly unlikely to interact with each other. The cellular localization information was downloaded from the TAIR database. The dataset with the positive set and the negative set constructed in this way is named as PPI_loc._ Another one is to randomly pair proteins in the positive set and then exclude pairs that are already in the list of positive interacting pairs. The dataset with the positive set and the negative set constructed in this way is named as PPI_rand_. Both PPI_loc_ and PPI_rand_ included the equal number of interacting and non-interacting pairs; thus there are 9,518 pairs in total.

For training and testing RF, protein pairs that lack co-expression information needed to be removed. This reduced the number of interacting pairs to 3,427. By adding the equal number of non-interacting protein pairs either from PPI_loc_ or PPI_rand_, the total number of the dataset for RF has become 6,854. The dataset was were split into a training, a validation, and a testing set, respectively, and a rigorous nested cross-validation evaluation was performed to evaluate the prediction accuracy of PPIP.

### The design of PPIP

**Figure 1:**
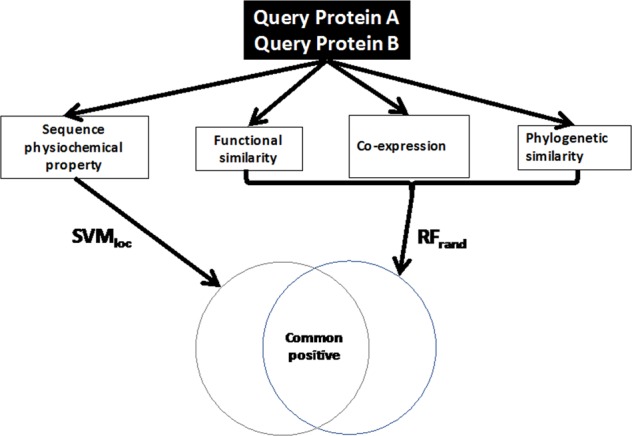
The schematic workflow of protein-protein interaction prediction by PPIP. Given a pair of protein A and B, physiochemical property features from their amino acid sequences are extracted and their interaction is predicted by support vector machine (SVM_loc_). In parallel, functional features including functional similarity scores, gene co-expression scores, and phylogenetic profile similarity score are used to make an independent prediction of interaction by random forest (RF_rand_).

PPIP predicts if a pair of proteins is likely to have physical interaction or not in physiological condition from the proteins’ sequence and proteomic features. As illustrated in Fig. 1, for a query protein pair, their physical interaction is predicted from physiochemical property features of amino acid sequences of the protein pairs using SVM. The SVM protocol was named as SVM_loc_ and SVM_rand_ corresponding to the PPI_loc_ and PPI_rand_ dataset used, respectively. The features used were hydrophobicity, hydrophilicity, side-chain volumes, polarity, polarizability, solvent-accessible surface area, and net charge index (NCI) of side-chains. In parallel, the complementary features of gene co-expression, functional similarity, and the phylogenetic profile^68^ were used to make another independent prediction by RF. The RF protocol was named as RF_loc_ and RF_rand_ corresponding to refer to the PPI_loc_ and PPI_rand_ dataset used, respectively. Predictions with RF were performed in two settings, one with all the features and the other without gene expression features (thus three functional similarity features and the phylogenetic profile) because gene expression data is currently not available for corn and soybean. See Methods for more details about the features.

### Prediction performance on the known *Arabidopsis* PPIs

On the PPI_loc_ and PPI_rand_ datasets of known PPIs and non-interacting protein pairs of *Arabidopsis*, parameters of SVM and RF were trained and tested using six-fold nested cross-validation. In this rigorous validation procedure, the dataset is split into six subsets, and prediction accuracy was measured on each of the subsets using parameters optimized on the rest of the five subsets. See Methods and Supplementary Tables S2 and S3 for more details of this procedure.

Using SVM, the overall prediction accuracies for the PPI_loc_ and PPI_rand_ datasets were 91.9% and 70.8%, respectively (Supplementary Table S4). Thus, SVM performed better on the negative set with protein pairs from different cellular locations than on negative protein pairs that were randomly combined from the interacting pairs (Supplementary Table S4). This is consistent with the conclusion in the paper by Guo^67^, who performed a similar comparison of negative datasets.

On the other hand, RF_rand_ trained on PPI_rand_ performed better than RF_loc_, which was trained on PPI_loc_. This order was consistently observed when the eight and the four features were used in RF. The accuracy of RF_8rand_ and RF_4rand_ were 92.0% and 92.6% accuracy, respectively. On the PPI_loc_, the accuracies were lower, 80.0% and 79.6% for RF_8loc_ and RF_4loc_, respectively (Supplementary Table S5).

An advantage of random forest is that it can provide the importance of each feature in making correct classification using two metrics, the mean decrease of accuracy (MDA) and the mean decrease of Gini importance (MDGI) (see Methods and Supplementary Note)^69^. As shown in Supplementary Table S6, two functional association scores, IAS, PAS, were found to be the two most important features for both RF_8loc_ and RF_8rand_ models. The IAS score was calculated based on the frequency of two GO terms annotating interacting proteins while the PAS score was calculated from the co-occurrence of two GO terms in PubMed abstracts. Therefore, it is reasonable that these two scores contribute largely to classifying interacting and non-interacting protein pairs, because they evaluate biological contexts of GO terms. The results in the table also showed that the phylogenetic profile was more informative than gene expression features.

### Comparison with STRING confidence scores

We compared our prediction with the confidence score of protein-protein interactions provided in the STRING database^50^. Since SVM used in the PPIP pipeline perform binary classifications and do not provide probability values that are comparable to the STRING scores, we used SVM regression for this comparison. Two types of SVM regression models were used, SVM-${\epsilon }$ and SVM-$\upsilon$^70^. SVM-${\epsilon }$ controls the error tolerance in the training set whereas SVM-$\upsilon$ uses an additional parameter $\upsilon$ to control the proportion of the data points to be used as support vectors. We trained these two SVM regression models using the same hyperparameters as SVM_loc_ and SVM_rand._ As for RF, we used the fraction of decision trees in RF that vote for protein-protein interaction as the probability.

First, we checked how consistent the predictions by SVM-ε and SVM-ν were with the SVM models in PPIP if a probability value of 0.5 in the SVM regressions was used to convert a probability value of SVM-ε and SVM-ν to binary prediction. On the PPI_loc_ dataset, the prediction by SVM_loc_-ε was consistent with SVM_loc_ on 99.2% (9445 among 9518) of the cases while SVM_loc_-ν was consistent with SVM_loc_ on 97.8% of the cases (9312 among 9518). On the PPI_rand_ dataset, SVM_rand_-ε’s results were consistent with SVM_rand_ on 73.9% (7032 among 9518) of the cases while SVM_rand_-ν’s results agreed with SVM_rand_ on 73.4% (6987 among 9518) of the proteins.

**Figure 2:**
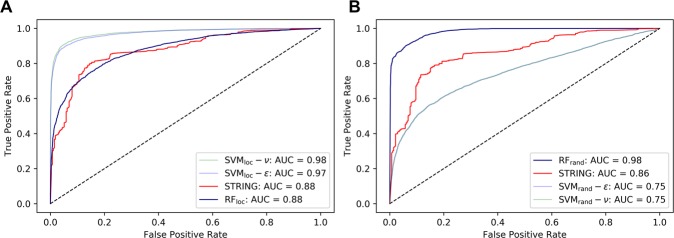
Comparison of PPI detection performance with STRING in identifying interacting protein pairs in *Arabidopsis*. Area Under the Curve (AUC) of Receiver operating characteristic (ROC) was used for comparison. (**A**) Evaluation on the PPI_loc_ dataset; (**B**) Evaluation on the PPI_rand_ dataset. SVM_loc/rand_-ε and –ν are SVM regression models trained on the PPI_loc_ or PPI_rand_, respectively. The probability for RF was computed as the fraction of votes from decision trees. For STRING, protein pairs were not included if they are not listed in STRING. A dashed line shows a random retrieval, which has an AUC of 0.5.

With these agreement results, we then compared the performance of SVM-ε, SVM-ν, and RF with STRING. The two datasets, PPI_loc_ and PPI_rand_, were used, and the comparison was made using Area Under the Curve (AUC) of Receiver operating characteristic (ROC) (Fig. 2). On the PPI_loc_ dataset (Fig. 2A), two SVM models, SVM_loc_-ε and SVM_loc_-ν showed substantially higher AUC (0.98 and 0.97, respectively) than STRING. RF showed the same AUC value as STRING (0.88). On PPI_rand_, RF_rand_ performed the best with AUC of 0.98 and STRING came the second (AUC: 0.86) (Fig. 2B). Thus, on the both datasets, at least one of our methods showed substantially better AUC than STRING.

Based on these results (Fig. 2), we combine SVM_loc_ and RF_rand_ in the PPIP pipeline (Fig. 1) since they achieved a very high AUC, substantially better than STRING, in the genome-scale prediction in *Arabidopsis*, corn, and soybean.

### PPI detection by taking overlap between SVM and RF

In Supplementary Table S7, we examined PPI detection performance by combining SVM and RF on the two datasets, PPI_loc_ and PPI_rand_. As shown in the table, precision improved by taking consensus: On PPI_loc,_ while the precision of SVM_loc_ and RF_loc_ were 0.947 and 0.823, respectively, the combination of the two methods showed a higher value of 0.980. Similarly, On PPI_rand,_ the combination of the two methods showed the perfect precision of 1.0. Note that the improvement of precision was a tradeoff with the accuracy, which decreased because apparently PPIs are missed if they are not detected by both SVM and RF. However, we consider that maintaining a high precision is more important when it comes to a genome-scale prediction because producing many false positive would be a serious concern.

We have further tested the performance of SVM_loc_ and RF_rand_ on a large negative dataset of 2,038,222 protein pairs that are assembled by exhaustively pairing proteins from different cellular locations. The false positive rate of SVM_loc_ on this dataset was 63.7%. We also ran RF_rand,_ after excluding pairs that do not have gene expression data (so that the RF model can run with gene expression features), which remained 1,048,575 pairs in the dataset. RF_rand_ recorded a small false positive rate of 4.36%. Finally, as designed in the PPIP pipeline, we took the overlap between SVM_loc_ and RF_rand_, which yielded a very small false positive rate of 2.68%. The results confirm that the design of PPIP was effective in reducing false positives and that PPIP is suitable for a genome-scale prediction.

### Prediction performance on the BioGRID *Arabidopsis* Dataset

We further tested the prediction ability of our prediction method PPIP on a different *Arabidopsis* dataset, which was obtained from the BioGRID database^29^. BioGRID contained PPI data that were not included in the TAIR database, partly because it was updated more recently. In total, 3,280 *Arabidopsis* physical PPIs which have been verified by at least two experiments and not included in the TAIR-based dataset were found in BioGRID. The data were further pruned by the sequence identity cutoffs, 80%, 50%, and 30% (Table 2). The overlaps between the training dataset (PPI_loc_ and PPI_rand_) were excluded. The sequence identity is a measurement of the protein sequence similarity from BLAST. Prediction by RF was applied for a smaller fraction of PPIs, only to those which have co-expression information. The data sets used by SVM and RF with different sequence identify cutoffs are provided in Supplementary Table S8. While an evaluation on over-prediction of our methods was extensively performed on a large negative dataset in the previous section, this benchmark provides an additional check of the SVM and RF models in terms of recall.

**Table 2:** The prediction accuracy (recall) on the BioGRID PPI dataset.

Seq. Identity cutoff	PPIs subject to prediction by SVM (RF)^(a)^	Recall by SVM_loc_^(b)^	Recall by RF_loc_^(c)^	Recall by SVM_rand_^(b)^	Recall by RF_rand_^(c)^
All PPIs	3280 (2468)	0.7466 (0.7057)	0.8440 (0.8327)	0.3250 (0.2565)	0.9444 (0.9444)
80%	1123 (797)	0.7266 (0.7114)	0.7804 (0.7604)	0.2654 (0.2597)	0.9448 (0.9448)
50%	937 (660)	0.7033 (0.6818)	0.7758 (0.7561)	0.2465 (0.2303)	0.9470 (0.9470)
30%	825 (585)	0.6909 (0.6667)	0.7641 (0.7453)	0.2364 (0.2239)	0.9470 (0.9470)

The PPIs were clustered by the sequence identity cutoffs of 80%, 50%, and 30% to reduce similar sequences. The sequence identity of protein pairs was computed with the Needleman-Wunsch (global sequence alignment) algorithm implemented in the nwalign python library. On this dataset, we evaluated recall, i.e. the fraction of PPIs in the datasets that were correctly predicted as interacting protein pairs. SVM trained by PPI_loc_ or PPI_rand_ were named as SVM_loc_ or SVM_rand_. RF trained by PPI_loc_ or PPI_rand_ were named as RF_loc_ or RF_rand_. (a) RF with the eight features was able to be applied only for PPIs that have gene co-expression data available. The numbers in the parentheses count such PPIs with expression data available. (b) Recall is the fraction of PPIs that are correctly predicted. In the parentheses, SVM recall values measured on the PPIs with co-expression data i.e. the same dataset as used for prediction by RF with the eight features, are shown. (c) The values show the recall of RF using the eight features including the gene expression features. Results with the four feature combinations that only use the functional association scores and the phylogenetic profile are provided in the parentheses.

The recall values of SVM were somewhat lower than what was observed on the TAIR-based dataset (0.8880 for SVM_loc_ and 0.5606 for SVM_rand_, which can be computed as the fraction of the sum of “True Positives” from the 1–6 test sets among the total of positives, i.e. the sum of True Positives” and “False Negatives” in Supplementary Table S4). On the other hand, the recall values of RF were in the same range as the value observed on the TAIR-based dataset (0.7642 for RF_8loc_, 0.7610 for RF_4loc_, 0.8984 for RF_8rand_ and 0.9050 for RF_4rand_ from Supplementary Table S5, which can be computed in the same way). RF’s two predictions with different feature sets, the full features and the feature set without gene expression features, yielded almost identical recall.

Among the two SVM models, SVM_loc_ showed a higher recall. When the two RF models were compared, RF_rand_ achieved a higher recall than the counterpart, RF_loc._ These results are consistent with what we observed on the TAIR-based dataset (Supplementary Tables S4 and S5), which would justify our earlier choice of combining SVM_loc_ and RF_rand_ for the genome-scale PPI predictions to be discussed in the subsequent sections.

### PPI prediction for three plant genomes

Next, we applied PPIP to the three plant genomes, *Arabidopsis*, *Zea mays* (corn), and *Glycine max* (soybean). The genome sequences of the three plants were downloaded from the UniProt database^71^. Since the number of all possible protein pairs in the whole genome is too large, we applied PPIP only for protein pairs that are likely to co-locate in a cell, having a sufficient similarity in their Cellular Component (CC) Gene Ontology (GO) category terms^72^, which describe the sub-cellular locations of proteins. Since many protein genes in corn and soybean do not have GO term annotations in UniProt, we used a function prediction method, PFP^73–75^ to predict GO terms to supplement annotations to proteins. PFP is one of the top performing function prediction methods, which performs better than conventional methods, e.g. BLAST^76^, as was also demonstrated in a community-wide function annotation assessment, CAFA^77,78^. From PFP, only high confidence GO predictions with a score of over 10,000 were used^73^. The similarity of CC terms of two proteins was evaluated by the FunSim score, which essentially is the average pairwise similarity of CC GO terms^79,80^. Protein pairs with a FunSim score of CC terms over 0.4 were subject to the prediction with PPIP. This cutoff was determined from the distribution of the CC-FunSim score of predicted *Arabidopsis* PPIs in three previous papers that made predictions based on the assumption that PPIs are conserved across species^42,61,64^ (Supplementary Fig. S1). Proteins that do not have CC annotations even with PFP prediction were also discarded from the PPI prediction. Applying this pre-screening reduced the number of protein pairs to 21.36% (133,074,361 pairs), 13.54% (24,814,793 pairs), and 15.27% (54,814,995 pairs) of all possible protein pairs for *Arabidopsis*, corn, and soybean, respectively. This pre-screening process would likely miss some true PPIs that do not satisfy the CC similarity criteria, nevertheless, we decided to apply the process because having a common subcellular co-localization can serve as additional supporting evidence of PPIs.

The numbers of predicted PPIs in the three genomes by PPIP are summarized in Table 3. For protein pairs that satisfied the CC GO term similarity, SVM_loc_ and RF_rand_ were independently applied, and commonly predicted PPIs by SVM_loc_ and RF_rand_ were selected. Among protein pairs with CC-FunSim > 0.4, SVM_loc_ selected about 10%, 56% and 56% as interacting pairs while RF_rand_ predicted 1.83%, 17.45%, and 18.06% as interacting, for *Arabidopsis*, corn, and soybean, respectively (Table 3). The final PPI predictions, which are the PPIs predicted commonly by SVM_loc_ and RF_rand_, were 0.0081%, 7.19%, and 3.77% out of all the possible protein pairs for *Arabidopsis*, corn, and soybean, respectively. Compared to the fraction of known PPIs in several other well-studied organisms in Supplementary Table S9, the fraction of *Arabidopsis*, corn, and soybean PPIs from the current study is at the same level. Particularly, the fraction of predicted PPIs for *Arabidopsis* (0.0081%) seems relatively small, but this fraction is consistent in Table S9. All the predicted PPIs for the three plant genomes are available as Supplementary Data on our lab website (http://kiharalab.org/PPIP_results/).

**Table 3:** The Summary of the number of predicted PPIs in the three plant genomes.

Organism	CC > 0.4	SVM_loc_	RF_rand_	Common	Degree exponent
*Arabidopsis*	133,074,561 (21.36%_all_)	13,682,168 (10.28%_cc_)	2,440,139 (1.83%_cc_)	50,220 (0.0081%_all_)	1.362
corn	24,814,793 (13.54%_all_)	13,902,459 (56.02%_cc_)	23,223,947 (17.45%_cc_)	13,175,414 (7.19%_all_)	0.204
soybean	54,814,995 (15.27%_all_)	30,844,273 (56.27%_cc_)	24,031,016 (18.06%_cc_)	13,527,834 (3.77%_all_)	0.401

**Figure 3:**
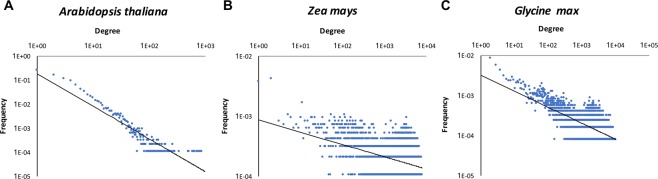
Degree distribution of proteins in the predicted protein-protein interaction network. The X-axis is the degree of proteins in the PPI network and the Y-axis is the frequency of proteins with a certain number of degrees. A log scale is used for both axes. (**A**) *Arabidopsis thaliana*; (**B**) *Zea mays* (corn); (**C**) *Glycine max* (soybean). The exponents of power-law distribution are 1.362, 0.204, 0.401, respectively.

CC > 0.4, the number and the percentage of protein pairs among all the possible protein pairs that satisfied the CC FunSim score criterion of over 0.4; (%_all_); SVM_loc_ and RF_rand_, predicted PPIs among pairs that satisfied the CC > 0.4 criterion by SVM_loc_ and RF_rand_, respectively; Common, commonly predicted PPIs by SVM_loc_ and RF_rand_; Degree exponent, the parameter value of the power-law distribution of PPIs (Fig. 3). %_all_ is the percentage relative to the all possible protein pairs of the organism while %_cc_ is the percentage relative to the protein pairs that satisfied CC > 0.4.

It is known that the degree distribution of a PPI network of an organism follows a power-law distribution, i.e. the histogram of the number of interactions (called the degree) for each protein is well approximated with a power-law, *p*(*k*) *~ k*^*−γ*^ where *k* is the fraction of proteins with a certain number of interactions, and *γ* is a parameter called the degree exponent, which determines the slope of the distribution^79,81,82^. Figure 3 shows that the PPIs of the three plants detected in the current work follow the power-law, with *γ* being 1.362 in *Arabidopsis*, 0.204 in corn, and 0.401 in soybean. Smaller degree exponents for corn and soybean indicate that these two plants have more hub proteins that interact with many proteins.

Next, we compared our PPI prediction on *Arabidopsis* with three existing genome-scale prediction results. These three works used a very different approach for prediction, the interlog concept^39^, which assumes that interactions of orthologous proteins across different species are conserved. Geisler-Lee *et al*.^61^ and De Bodt *et al*.^42^ used the same reference organisms, *S*. *cerevisiae*, *C*. *elegans*, *D*. *melanogaster*, and *H*. *sapiens*, whereas Dutkowski *et al*.^64^ used the same four organisms with two more organisms, *M*. *musculus*, and *R*. *norvegicus* (Supplementary Table S10). Supplementary Table S11 shows the number of PPIs predicted by the three works. All the three works predicted about the same number of PPIs, 14,009 to 19,974. The works by Geisler-Lee *et al*. and De. Bodt *et al*. made a very similar number of predictions, which is probably due to them using the same set of reference organisms. In Supplementary Table S11, we compared commonly predicted PPIs by pairs of works including our method, PPIP. Geisler-Lee *et al*. and De. Bodt *et al*. had the largest number of common predictions, although the common predictions would be small considering the same approach and the reference organisms they used. Common predictions by other pairs including pairs with PPIP are roughly about the same numbers. Thus, PPIP has a similar level of agreement with the three previous works, although they took a very different approach from ours.

### Examples of predicted PPIs in *Arabidopsis*

From the predicted PPIs for the three plant genomes (http://kiharalab.org/PPIP_results/), here we discuss examples with three different levels of confidence. Supplementary Table 12–21 provide examples from *Arabidopsis*. All the listed PPIs in the tables were predicted consistently by the SVM_loc_ and RF_rand_ and the probability score of RF_rand_ was over 0.95. The difference of the confidence levels is based on the availability of additional supporting data.

The predictions are separated into three classes for each genome according to the availability of other evidence that supports the predictions. Supplementary Table S12 lists predicted PPIs with two more supporting evidence: a very high score of over 900 in the STRING database^50^ and also satisfy at least one of the following three conditions: (a) the two proteins are known to locate in the same pathway in the KEGG database^83^, (b) the two proteins are co-mentioned in a literature. The predicted PPIs with correlated elution profile in PPI detection using mass spectrometry (MS)^84,85^ are also included in Supplementary Table S12. STRING collects several different types of evidence for the functional association of protein pairs and provides a score that ranges from 0 to 1000 with 1000 as the most confident score. In the works by Aryal *et al*.^84,85^, proteins in *Arabidopsis* were fractionated using size exclusion chromatography, and abundance profiles across the column fractions were quantified using label-free precursor ion (MS1) intensity. Proteins with correlated profiles and clustered together among all detected proteins were more likely to form complexes (see the original papers for more details). Supplementary Table S13 lists predicted PPIs with at least one piece of additional evidence, either a common KEGG pathway or literature that co-mention the two proteins. Protein pairs listed in the Supplementary Table S14 do not have additional evidence because the proteins were not much studied before but were predicted with the highest score (1.0) by RF_rand_.

The first half of Supplementary Table S12 lists predicted *Arabidopsis* PPIs with literature that describes evidence of their interaction. This list selected the most confident prediction in the *Arabidopsis*. Most of the interacting proteins are ribosomal proteins. Besides ribosomal proteins, several pairs of Sm-like proteins are predicted to interact, which are known as subunits of the heteroheptameric complexes and function in mRNA splicing and degradation^86,87^. Another predicted PPI is plastid division protein PDV2 (AT2G16070) and protein accumulation and replication of chloroplasts 6 ARC6 (AT5G42480), which has been shown to interact in the intermembrane space to coordinate the division machinery of chloroplast membrane^88^. Our predicted PPIs also included some subunits of known complexes such as coatomer, RNA polymerase, DNA directed RNA polymerase, augmin, and adaptor complex 1 (AP-1).

The latter half of the table provides PPIs with similar MS elution profiles^84,85^. For example, V-type proton ATPase subunit C (AT1G12840) and V-type proton ATPase subunit G2 (AT4G23710) are predicted to interact by RF and SVM. Additionally, they have highly correlated protein elution profile and are involved in the two common KEGG pathway including oxidative phosphorylation (ath00190), and phagosome (ath04145).

Supplementary Table S13 lists predicted PPIs in Arabidopsis with the next level of confidence, which has extra evidence but no information available in STRING or with a low STRING score (<400). An interesting example is asymmetric leaves 2 (AS2) (AT1G65620) and histone deacetylase 6 (HDA6) (AT5G63110). Although they have a very low STRING score a previous study showed that HDA6 functions with AS2 to regulate the leaf development and suggested that HDA6 may be the part of the AS1-AS2 repression complex to repress *KNOX* gene expression in *Arabidopsis*^89^. Another interesting example is protection of telomeres protein 1a POT1a (AT2G05210) and CST complex subunit TEN1 (AT1G56260). It is known that CTC1, STN1, and TEN1 consist of telomere complex and POT1a interplay with CST components to regulate the telomerase enzyme activity^90^.

The last table for *Arabidopsis*, Supplementary Table S14, shows a list of PPIs with the highest RF probability score yet have no other known evidence. An interesting example in this table is pentatricopeptide repeat-containing protein (AT1G05600 and AT5G27270). They may function in RNA editing in chloroplast^91^.

### Examples of predicted PPIs in corn

**Figure 4:**
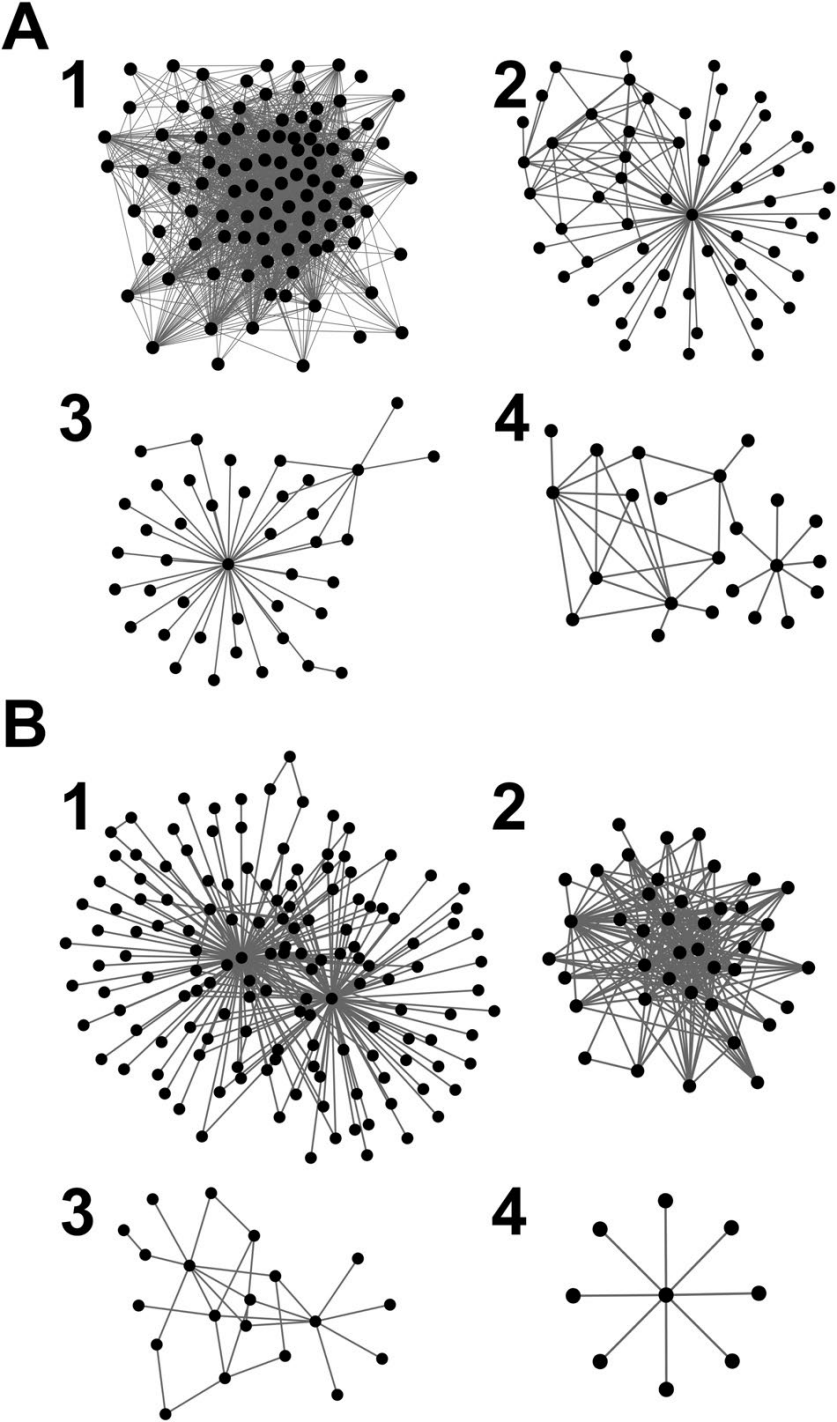
The networks constructed with predicted PPIs with the highest RF confidence scores (1.0) but do not have documented other supporting evidence. Connected PPIs are predicted by both SVM_loc_ and RF_rand_. PPIs were further selected by high functional similarity scores: IAS-FunSim (>200), PAS-FunSim (>20), and the phylogenetic profile similarity (>0.9). (**A**) Predicted PPIs for corn. 224 out of 226 proteins in the PPIs qualified for the criteria are included in four subnetworks shown. The number of proteins in each network is 101, 59, 41, and 23 for the subnetwork 1 to 4, respectively. Supplementary Table S17 provides the subnetwork index of each predicted PPI. (**B**) Predicted PPIs for soybean. 215 out of 224 proteins in the PPIs qualified for these criteria are included in four subnetworks shown. The number of proteins in each network is 145, 41, 20, and 9 for the subnetwork 1 to 4, respectively. Supplementary Table S21 provides the subnetwork index of each predicted PPI. See Supplementary Table S18 for the results of the functional enrichment analysis of the subnetworks.

Predictions for corn are shown in Supplementary Tables S15–S17, and Fig. 4A. PPIs shown in these tables are predicted both by the SVM_loc_ and RF_rand_ with a high RF probability score of 0.9 or higher. As in the tables for *Arabidopsis*, Supplementary Table S15 lists predicted PPIs with two additional supporting pieces of evidence, and Supplementary Table S16 is for PPIs with a single existing piece of additional evidence, in this case having a common KEGG pathway, while Supplementary Table 18 includes the predicted PPIs with no existing evidence for interaction.

In Supplementary Table S15, 12 protein pairs out of 18 listed turned out to be NAD(P)H-quinone oxidoreductase subunits. This list also includes interaction between hydroxymethylglutaryl-CoA synthase (UniProt ID: B6U9M4) and acetyl-CoA acetyltransferase (UniProt ID: B4F9B2), both of which are involved in the four same pathways including terpenoid backbone biosynthesis (zma00900), valine leucine and isoleucine degradation (zma00280), butanoate metabolism (zma00650), and synthesis and degradation of ketone bodies (zma00072), and they have a very high database score in STRING.

Supplementary Table S16 shows predicted PPIs with the highest RF score (1.0) locating in at least one common KEGG pathway. For these PPIs, a STRING score was not available. As shown, most of the proteins are kinases in the same pathways, the plant hormone signal transduction (KEGG: zma04075) or the plant-pathogen interaction pathway (KEGG: zma04626). Thus, it is reasonable to conclude that they are interacting. In the table, we also provided protein functional association score (FunSim) with IAS scores. As mentioned in the Methods, IAS directly indicates the likelihood that proteins with the GO annotations interact. Since the IAS scores listed in the table are very high relative to the background distribution (within top 1% for IAS and PAS for proteins in *Arabidopsis* protein pairs), these scores also support the PPI predictions.

The third list, Supplementary Table S17, includes PPIs that were predicted with a high confidence score (RF probability = 1) and high functional correlations (IAS > 200 and PAS > 20 and phylogenetic similarity >0.9), but do not have other existing supporting information or protein annotations. When the 226 proteins involved in these PPIs were represented into a network by connecting protein pairs in the PPIs using Cytoscape^92^, 224 of them (99.1%) are clustered into four subnetworks (Fig. 4A). Since they have no functional annotations, we used PFP to predict their GO terms and performed GO enrichment analysis using NaviGO^80,93^ as shown in Supplementary Table S18. It turned out that each of the subnetworks has enriched GO terms that are common in the proteins in the network: The largest subnetwork involved in 101 proteins were predicted by PFP to be involved in flavonoid glucuronidation, flavonoid biosynthetic process, cellular glucuronidation, and quercertin 3-O-glucosyltransferase activity. In the second largest subnetwork, 57 out of 59 proteins were predicted to be involved in the RNA metabolic process and RNA secondary structure unwinding. All proteins in the third subnetwork were predicted to function in proteolysis and protein catabolic process while proteins in the last subnetwork were predicted to be involved in the regulation of the metabolic process, regulation in gene expression, and regulation of transcription DNA-templated. Thus, proteins in the predicted PPI networks have coherent biological functions.

### Examples of predicted PPIs in soybean

The last plant genome we analyzed was soybean. Soybean has much less available functional information in databases comparing with *Arabidopsis* and corn. Supplementary Table S19 selected a list of predicted PPIs using the same standard as Supplementary Table S15 for corn, i.e. PPIs supported by two additional evidence. This list includes subunits from known complexes, including ATP synthase, chalcone synthase, cytochrome, and NADH dehydrogenase.

The next table, Supplementary Table S20 shows PPIs without conclusive information in STRING. However, two proteins in each pair are found in the same KEGG pathway, and most of them have a similar function, judging from the name in KEGG or UniProt annotation. The predicted PPIs in this list include protein interaction between glycosyltransferase and protein kinase involved in plant hormone signal transduction, plant-pathogen interaction, zeatin biosynthesis, and carotenoid biosynthesis signaling pathway.

Supplementary Table S21 lists high confident PPIs (RF probability = 1, IAS > 200, PAS > 20 and phylogenetic similarity >0.9), which do not have other existing supporting evidence and functional annotation in UniProt. As we did for the predicted PPIs in corn, in Fig. 4B we constructed networks with 224 proteins that are involved in the PPIs in Table S21 and performed the functional enrichment analysis using predicted GO terms by PFP (Supplementary Table S18, the bottom half). 215 (96.0%) out of 224 proteins were included in four subnetworks. As observed in the subnetworks in corn (Fig. 4A), proteins in each subnetwork are highly functionally relevant and would be reasonable to conclude that they are most likely to interact. All proteins in the largest subnetwork were predicted to be involved in the MAPK signaling pathway in response to stimuli. The second largest subnetwork with 41 proteins was predicted to be involved in the flavonoid biosynthetic process. Proteins in the third subnetwork are predicted to be involved in RNA processing and intracellular protein transport. The fourth subnetwork with 9 proteins is in a pathway for signal transduction and cell communication in response to the stimulus.

## Discussion

A PPI network is fundamental for understanding an organism’s functional and structural units. For example, PPIs are very useful for predicting the function of individual proteins^3^ as well as pathways of protein groups^94^. Although large-scale PPIs of several model organisms have been revealed by experimental methods^95–97^ and by computational methods^42,61,64,98^, the works for plant PPIs were sparse. This work is intended to fill the gap for plant PPIs by providing PPI predictions with the method that was calibrated on known PPIs in *Arabidopsis*.

It is inevitable that a computational method often makes wrong PPI predictions. However, as discussed in the introduction, the situation would be similar in experimental methods, as it has been reported that independent experiments have substantial disagreements^26–28^. To reduce errors of a method, either computational or experimental, it would be useful to compare outputs from multiple methods. Having this idea in mind, we designed PPIP such that it combines two independent predictions, one using sequence-based features and the other with a combination of orthogonal features (Fig. 1). This architecture sacrificed the recall rate but in return achieved a very low false positive rate, which we consider as a higher priority since we provide all predicted PPIs for reference information for biologists. Also, in the genome-scale predictions for the three genomes, we provided additional evidence from other sources whenever available. In the analysis, we highlighted predicted PPIs with three levels of confidence. All the PPIs in these three levels were predicted not only with high scores but with additional supporting evidence. PPIs in the first (best) confidence have direct literature information or multiple supporting data, including a high score in STRING and co-existence in the same pathway. In the second level, PPIs have at least one evidence including the co-existence in the same pathway. PPIs of the third level confidence are between proteins with functional coherence. While it is true that functional similarity between proteins does not necessarily indicate physical interaction between them, it is certainly highly related with each other as it is a common practice to verify experimentally detected PPIs by checking their functional similarity^17,18,96^ and functional similarity is an informative feature of proteins for predicting PPIs^45,46,99–101^. PPI predictions made for the three plants are made available on our lab website (http://kiharalab.org/PPIP_results/). We hope they are used as a reference and found informative.

## Methods

Below we describe details of features and the machine learning methods used to predict PPIs.

### Sequence-based prediction

We explain protein sequence features used in PPIP. It is the left branch of the flowchart in Fig. 1. To capture physicochemical properties of interacting proteins the following seven features are assigned to each amino acid of query protein sequences^102–108^: hydrophobicity, hydrophilicity, side-chain volumes, polarity, polarizability, solvent-accessible surface area, and net charge index (NCI) of side-chains. Then each query protein sequence is represented with auto-covariance (AC) using strings of the seven features as follows:

1 $$AC(lag,j)=\frac{{\sum }_{i=1}^{L-lag}({P}_{i,j}-\frac{1}{L}{\sum }_{i=1}^{L}{P}_{i,j})\times ({P}_{(i+lag),j}-\frac{1}{L}{\sum }_{i=1}^{L}{P}_{ij})}{L-lag}$$

where *lag* is the distance between covariant residues to consider, which ranges from 1 to 30, *j* is the *j-th* physiochemical feature, *i* is the position in the sequence, P*i*,*j* is the value of the physicochemical feature *j* of amino acid position *i*, and *L* is the length of the sequence. Thus, AC of a physicochemical feature with a certain *lag* length will be large if amino acid with a large (or small) property value appears periodically with an interval of *lag*. AC is computed for each protein sequence, and thus a query protein pair is represented as a 2 (sequences) * 7 (features) * 30 (lag intervals) = 420-dimensional vector. The vector representation was used as input of SVM for predicting if protein pairs are interacting or not interacting. We took this approach because it was reported to be successful in a previous paper^67^.

### Gene expression features

On the other branch of PPIP (Fig. 1), we used three features, gene co-expression, functional similarity, and phylogenetic profile similarity in the framework of RF to predicted PPI of a query protein pair. We explain the three features in the following three subsections.

If two proteins are upregulated or down-regulated simultaneously under various conditions, it is highly likely that the two proteins are involved in the same pathway and have a higher chance that they physically interact with each other. Thus, co-expression patterns can provide indirect evidence for predicting PPIs. We obtained gene coexpression information derived from microarray experiments and RNA-seq experiments from the ATTED-II database (http://atted.jp/)^109^. It is a database of pre-calculated Pearson’s correlation coefficients (PCC) and the mutual rank (MR) of co-expressed genes. MR is defined as the geometric mean of the rank of the correlation gene A to gene B among proteins in the genome and the rank of gene B to gene A. The smaller MR is, the stronger the genes are co-expressed. Since gene expression data are provided in two sources, microarray and RNA-seq, four features (microarray MR, microarray PCC, RNA-seq MR, and RNA-seq PCC) were used to represent the co-expression profile of protein pairs.

### Protein function features

The second feature used is protein functional similarity. Proteins with the same or similar biological functions are likely to physically interact because they may form permanent complexes or take part in the same pathway. Functional similarity of proteins was quantified by established similarity scores of Gene Ontology (GO) terms^72^. GO annotations were obtained from UniProt^71^ and TAIR (for *Arabidopsis*). We used three GO similarity/relevance scores, Interaction association score (IAS)^45^, Co-Occurrence Association Score (CAS), and PubMed Association Score (PAS)^80,93,110^. IAS, CAS, and PAS quantify how significantly a pair of GO terms appear in physically interacting proteins, annotations of individual genes, and PubMed abstracts. Thus, they evaluate co-occurrence of GO terms in biological contexts and shown to be effective in identifying proteins that physically interact)^45^ or in the same pathways^111^.

### Phylogenetic profile similarity

It has been observed that interacting proteins tend to coevolve^112^. We use the phylogenetic profile to exploit the evolutionary co-occurrence patterns of interacting proteins. The basic assumption of the phylogenetic profile method is that interacting proteins either co-present or co-absent across organisms^68^. The original phylogenetic profile^68^ is a binary pattern of presence or absence of homologs in a set of reference genomes, but we used a modified version of the profile that used BLAST bit score instead of binary values as follows^113^:

2 $$sim(i,j)=\frac{{\sum }_{k=1}^{n}{R}_{ik}\times {R}_{jk}}{{[({\sum }_{k=1}^{n}{R}_{ik}^{2})\times ({\sum }_{k=1}^{n}{R}_{jk}^{2})]}^{1/2}}$$

where

3 $${R}_{ik}=\frac{{B}_{ik}}{{B}_{ii}}$$

The similarity of protein *i* and *j* are defined in Eq. 2. *k* is the *k*-th reference genome, ${R}_{ik}$ is the BLAST search bit score of homolog of protein *i* in the *k*-th genome divided by the BLAST search bit score of *i* in the query genome (Eq. 3). We used 100 reference genomes (*n* = 100) (Supplementary Fig. S2). These genomes were selected in the following steps: we ran BLAST searches from all *Arabidopsis* protein sequences against the UniProt database using the default E-value cutoff of 10. Then, we constructed a phylogenetic tree for the genomes and manually selected the genomes from each branch of the tree so that the selected genomes are well distributed and represent the tree.

### Machine learning methods

We used two machine learning methods in PPIP, SVM for making predictions from sequence features and RF for predicting from a combination of four other features (Fig. 1). For SVM, we used the software package libsvm 2.84^114^. SVM uses a kernel function to transform input features and two hyper-parameters that need to be determined, a regularization parameter γ, which defines how far each training data influences the model and *C*, which controls the tradeoff of misclassification on training examples. For our kernel function, we used a radial kernel following previous works that predict PPI prediction from protein sequence features^38,115–117^. The two hyper-parameter values, *C* and *γ*, were determined to be $lo{g}_{2}C=5$ and $lo{g}_{2}\gamma =-\,1$ for SVM_loc_ and $lo{g}_{2}C=1$ and $lo{g}_{2}\gamma =1$ for SVM_rand_ by performing nested cross-validation^118,119^ as shown in Supplementary Table 2.

In parallel to the sequence-based prediction with SVM, RF was used to make an independent prediction from three features, functional similarity, gene co-expression, and phylogenetic profile similarity. RF is an ensemble learning method, which combines predictions made by a number of decision trees by a majority vote. RF can also determine important variables that contributed most in classification by calculating two metrics, the mean decrease of accuracy (MDA) and the mean decrease of Gini importance (MDGI) (refer to Additional file 8: Note S1 for more details)^69,120^. MDA is the difference of the error rate of classification caused by permuting feature values with values of other data points in a dataset. MDGI tells how much less a particular feature is selected as a node in the random forest after permuting this feature. The larger MDA and MDGI of a certain feature are, the more important that feature is. Similarly to SVM, we performed nested cross-validation to determine three hyper-parameter values used in RF (Supplementary Table S3).

## Supplementary information

Supplementary Information

Supplementary Table S1

Supplementary Table S8

Supplementary Table S12, S13, S14

Supplementary Table S15, S16, S17

Supplementary Table S19, S20, S21

## Data Availability

All data generated or analyzed during this study are included in this article and its supplementary information files. The genome-scale prediction results are available on our lab website http://kiharalab.org/PPIP_results/.
