# Supplementary material for: Computational identification of protein-protein interactions in model plant proteomes
Source: Sci Rep. 2019 Jun 19;9:8740. doi: 10.1038/s41598-019-45072-8 (PMC6584649; doi:10.1038/s41598-019-45072-8)
Supplement: Supplementary file 1 — Supplementary Information [file 41598_2019_45072_MOESM1_ESM.pdf]

Supplementary information

Computational identification of protein-protein interactions in model plant proteomes

Ziyun Ding[1] and Daisuke Kihara [1, 2, 3*]

[1] Department of Biological Science, Purdue University, West Lafayette, IN, 47907 USA

[2] Department of Computer Science, Purdue University, West Lafayette, IN, 47907 USA

[3] Department of Pediatrics, University of Cincinnati, Cincinnati, OH, 45229, USA

* Corresponding author

Email: ZD: ding48@purdue.edu DK: dkihara@purdue.edu

Supplemental Table S1. (in a separate Excel file) The experimentally verified protein-protein interactions (PPIs) downloaded from TAIR database. Only the 4,776 PPIs identified by physical experimental systems are included. The PPIs only identified by genetic experimental systems are discarded and they are listed at the right side of the table highlighed in grey. After removing the protein length less than 50 residues, only 4,759 PPIs are used for training. The number in each cell indicates the number of the same type of experiment which identifies such PPI.

Supplementary Table S2. Prediction accuracy for validation sets from six-fold nested cross-validation on the *Arabidopsis* PPI dataset using SVM evaluated on the PPI_{loc} or PPI_{rand} datasets with a radial kernel.

**SVM_{loc}**	**c**	**g**	**Accuracy**
1	log2c= 5	log2g= -1	0.934
2	log2c= 5	log2g= -1	0.932
3	log2c= 5	log2g= -1	0.937
4	log2c= 5	log2g= -1	0.930
5	log2c= 5	log2g= -1	0.929
6	log2c= 5	log2g= -1	0.933
Average			0.932

To run SVM, two hyper-parameters, γ and *C*, need to be determined. From the *Arabidopsis* PPI dataset, short proteins whose length is less than 50 amino acids were excluded. The resulting dataset includes 4,759 interacting protein pairs and 4,759 non-interacting pairs. This dataset was separated into six subsets, where one subset was used for test and remaining five subsets were used for training and validation following the nested cross-validation procedure. By changing the test set among the six, the process was repeated six times. This corresponds to each row in the table.

In nested cross-validation, the five subsets were used further for five-fold cross-validation. Four out of the five subsets were used for training and one subset was used for validation of the trained model under each hyper-parameter combination. This was repeated five times using a different subset for validation, and a hyper-parameter combination that gave the best average accuracy over the five validation subsets was selected. The accuracy is calculated as the total number of corrected prediction (true positive and true negative) divided by the total number of data (including true positive, true negative, false positive, and false negative). The table shows the best hype-parameter combination found and the average accuracy obtained by the hyper-parameter combination for each of the test – training/validation separation. log_2C value was explored from -5 to 15 with a step size of 2 while $log_2\gamma$ was changed from 3 to -15 with a step size of -2. Thus, in total 11 * 10 = 110 combinations were examined.

For the PPI_{loc} dataset, $log_2C = 5$ and $log_2\gamma = -1$ were chosen because it was selected as the best for all six training/validation sets. In Supplementary Table S4, these hyper-parameters were used to retrain a SVM_{loc} model now using all five training/validation subsets, which was then applied to the test set.

Below is the results for the validation set using SVM_{rand}:

**SVM_{rand}**	**c**	**g**	**Accuracy**
1	log2c= 1	log2g= 1	0.768
2	log2c= 1	log2g= 1	0.765
3	log2c= 1	log2g= 1	0.775
4	log2c= 1	log2g= 1	0.766
5	log2c= 1	log2g= 1	0.760
6	log2c= 3	log2g= -1	0.774
Average			0.768

For the PPI_{rand} data set, the combination of $log_2 C = 1$ and $log_2 \gamma = 1$ was chosen because it was selected as the best in five out of six training/validation sets (a majority vote). In Supplementary Table S4, these hyper-parameters were used to retrain a SVM_{rand} model now using all five training/validation subsets, which was then applied to the test set.

Supplementary Table S3. Results of a six-fold cross-validation on the PPI_{loc} and PPI_{rand} dataset using random forest as the classifier.

There are two feature sets used: Eight features (RF_8), which used the mutual rank and Pearson's correlation coefficient calculated for gene expression from microarray experiments and RNA-seq data, IAS, PAS, CAS, and the phylogenetic profile similarity score, and four features (RF_4), which did not include gene expression data. Therefore, in total there are four combinations, considering two variations of the datasets and two feature sets.

The first table is on the PPI_{loc} dataset using eight features:

**RF_{8loc}**	**ntree**	**mtry**	**cutoff**	**Accuracy**
1	1400	5	0.5	0.796
2	1200	4	0.5	0.801
3	800	4	0.5	0.812
4	200	4	0.5	0.798
5	1400	3	0.5	0.790
6	600	5	0.5	0.798
Average				0.799

To run random forest, three parameters, ntree, mtry, and cutoff need to be determined (Please see the Supplemental Note 1 below). Four co-expression features, the Pearson correlation coefficients and mutual ranks from microarray data and RNA-sequencing data, three functional association scores, IAS, PAS, and CAS, and the phylogenetic profile similarity score were used as features. We performed nested cross-validation. Since, only 3,427 interacting protein pairs from the golden standard dataset have all eight features, we also randomly selected 3,427 non-interacting protein pairs to balance the dataset. The ntree value was explored from 200 to 1400 with a step size of 200, mtry was explored from 3 to 6 with a step size of 1, and the cutoff value was explored from 0.3 to 0.7 with a step size of 0.2.

For the PPI_{8loc} data set, the combination of ntree = 800, mtry = 4, and cut-off = 0.5 were chosen because this combination achieved highest accuracy among six training/validation sets. In Supplementary Table S5, these hyper-parameters were used to retrain the RF_{8loc} model, which was then applied to the test set.

The next table shows the results for the validation set of the PPI_{rand} set with eight features:

**RF_{8rand}**	**ntree**	**mtry**	**cutoff**	**Accuracy**
1	400	6	0.5	0.923
2	1200	6	0.5	0.923
3	400	4	0.5	0.924
4	400	6	0.5	0.910
5	400	6	0.5	0.915
6	1000	6	0.5	0.916
Average				0.919

The combination of ntree = 400, mtry = 6, and cut-off = 0.5 were chosen because it was selected as the best in three out of six training/validation sets (a majority vote). In Supplementary Table S5, these hyper-parameters were used to retrain the RF_{8rand} model and applied to the test set.

The third table is the results of a six-fold cross-validation on the PPI_{loc} dataset using the four features, IAS, PAS, CAS, and the phylogenetic profile similarity score.

**RF_{4loc}**	**ntree**	**mtry**	**cutoff**	**Accuracy**
1	400	2	0.5	0.793
2	200	2	0.5	0.794
3	800	2	0.5	0.808
4	1000	2	0.5	0.790
5	400	2	0.5	0.788
6	200	2	0.5	0.797
Average				0.795

The nested cross-validation was performed as in Supplementary Table S2. The ntree value was explored from 200 to 1000 with a step size of 200, mtry was explored from 2 to 4 with a step size of 1, and cutoff was explored from 0.3 to 0.7 with a step size of 0.2.

The combination of ntree = 200, mtry = 2, and cut-off = 0.5 were chosen for RF_{4loc} because this combination was selected in two out of six training/validation sets and achieved higher accuracy than ntree = 400, mtry = 2, and cut-off = 0.5.

The last table shows the results on PPI_{rand} using the four features:

**RF_{4rand}**	**ntree**	**mtry**	**cutoff**	**Accuracy**
1	400	2	0.5	0.931
2	200	2	0.5	0.930
3	600	3	0.5	0.928
4	1000	4	0.5	0.915
5	400	3	0.5	0.921
6	1000	2	0.5	0.923
Average				0.925

From the results, the combination of ntree = 400, mtry = 2, and cut-off = 0.5 was chosen because this combination achieved highest accuracy among six training/validation sets.

Supplemental Table S4. The prediction results on the dataset of known *Arabidopsis* PPIs using SVM.

Two results are shown. The first one is the results on the PPI_{loc} dataset (SVM_{loc}).

SVM_{loc}	Number of PPIs	True Pos.	True Neg.	False Pos.	False Neg.	Test Set Accuracy
1	1586	685	777	16	108	0.922
2	1586	717	707	86	76	0.898
3	1586	701	716	77	92	0.893
4	1586	721	742	51	72	0.922
5	1586	739	790	3	54	0.964
6	1588	663	791	3	131	0.916
Average						0.919

The results shown are the accuracy for each of the six subsets and the overall average of the known *Arabidopsis* PPI dataset used as the test set. The prediction was performed with SVM_{loc} using sequence-based features. The hyper-parameters used were log2c=5 and log2g=-1, which were determined in the nested cross-validation (Supplementary Table S2). True/False Pos., the number of true/false positives predictions; True/False Neg., true/false negative predictions.

The second results are on the PPI_{rand} dataset (SVM_{rand}).

SVM_{rand}	Number of PPIs	True Pos.	True Neg.	False Pos.	False Neg.	Test Set Accuracy
1	1587	353	698	97	440	0.662
2	1586	557	649	144	236	0.760
3	1586	470	676	117	323	0.723
4	1586	377	692	101	416	0.674
5	1586	482	679	114	311	0.732
6	1586	428	674	119	364	0.695
Average						0.708

The results shown are the accuracy for each of the six subsets and the overall average of the known *Arabidopsis* PPI dataset used as the test set. The prediction was performed with SVM_{rand} using sequence-based features. The hyper-parameters used were log2c=1 and log2g=1, which

were determined in the nested cross-validation (Supplementary Table S2). True/False Pos., the number of true/false positives predictions; True/False Neg., true/false negative predictions.

Supplemental Table S5. The prediction results on the dataset of known *Arabidopsis* PPIs using RF.

There are four results shown. The first two are on the PPI_{loc} dataset using RF with the eight features (RF_{8loc}) and four features (RF_{4loc}), respectively.

**With eight features**						
RF_{8loc}	**Number of PPIs**	**True Pos.**	**True Neg.**	**False Pos.**	**False Neg.**	**Test Set Accuracy**
1	1142	435	504	67	136	0.822
2	1142	430	460	111	141	0.779
3	1142	382	447	124	189	0.726
4	1142	438	483	88	133	0.806
5	1142	458	518	53	113	0.855
6	1144	476	453	119	96	0.812
Average						0.800
**With four features**						
RF_{4loc}	**Number of PPIs**	**True Pos.**	**True Neg.**	**False Pos.**	**False Neg.**	**Test Set Accuracy**
1	1142	433	498	73	138	0.815
2	1142	421	470	101	150	0.780
3	1142	399	437	134	172	0.732
4	1142	439	483	88	132	0.807
5	1142	450	498	73	121	0.830
6	1144	466	459	113	106	0.809
Average						0.796

Prediction on the PPI_{loc} dataset using RF was performed in two settings, one with eight features (RF_{8loc}), which include four gene expression-related features, three functional association scores, (IAS, PAS, CAS), and the phylogenetic profile, and the other with four features (RF_{4loc}), which are the three functional association scores and the phylogenetic profile. The hyper-parameters used were ntree=800, mtry=4, and cutoff=0.5 for RF_{8loc} and ntree = 200, mtry = 2, and cut-off = 0.5 for RF_{4loc}, both of which were determined in the nested cross-validation (Supplementary Table S3).

The next two are on the PPI_{rand} dataset using RF with eight (RF_{8rand}) and four features (RF_{4rand}):

**With eight features**						
RF_{8rand}	**Number of PPIs**	**True Pos.**	**True Neg.**	**False Pos.**	**False Neg.**	**Test Set Accuracy**
1	1142	482	545	26	89	0.899
2	1142	494	537	34	77	0.903
3	1142	479	543	28	92	0.895
4	1142	528	540	31	43	0.935
5	1142	549	535	36	22	0.949
6	1144	546	531	40	25	0.941
Average						0.920

**With four features**						
RF_{4rand}	**Number of PPIs**	**True Pos.**	**True Neg.**	**False Pos.**	**False Neg.**	**Test Set Accuracy**
1	1142	487	540	32	85	0.899
2	1142	493	536	36	79	0.901
3	1142	495	542	30	77	0.908
4	1142	534	550	22	38	0.950
5	1142	553	539	33	19	0.956
6	1144	544	536	36	28	0.944
Average						0.926

The hyper-parameters used were ntree = 400, mtry = 6, and cut-off = 0.5 for RF_{8rand} and ntree = 400, mtry = 2, and cut-off = 0.5 for RF_{4loc}, both of which were determined in the nested cross-validation (Supplementary Table S3).

Supplementary Table S6. The importance of variables for classifying positive and negative data using RF.

**RF_{8loc}**	**Negative**	**Positive**	**MDA**	**MDGI**
micro_MR	38.6121	22.57167	49.13145	187.8184
micro_PCC	29.49857	20.7819	38.0066	179.3132
RNA_MR	51.68748	21.52674	60.84656	214.8184
RNA_PCC	35.877	21.73235	47.67577	194.7815
IAS	118.53192	149.28542	198.01382	783.0412
PAS	88.13659	145.18851	181.64305	1096.2835
CAS	-11.86095	62.29549	50.44961	396.5665
PHY	49.57318	73.07908	81.35968	373.8518

**RF_{8rand}**	**Negative**	**Positive**	**MDA**	**MDGI**
micro_MR	9.692238	7.019082	10.45622	58.2901
micro_PCC	11.20431	8.213752	13.44388	58.13418
RNA_MR	20.07403	7.774314	13.95462	60.91505
RNA_PCC	12.833527	8.10697	14.92287	56.76476
IAS	126.754812	118.383743	170.353	1908.49263
PAS	50.476012	111.477796	99.08722	750.90133
CAS	53.036897	39.316735	55.15384	433.29671
PHY	6.013217	13.609914	13.19855	99.69469

micro_MR, micro_PCC, the mutual rank (MR) and the Pearson's correlation coefficient (PCC) from microarray data; RNA_MR, RNA_PCC, MR and PCC for RNA-sequencing data. IAS, PAS, CAS, three functional association scores. PHY, the phylogenetic profile similarity score. Negative, the mean decrease of the true negative rate in classifying non-interacting protein pairs; Positive, the mean decrease of the true positive rate in classifying interacting protein pairs. Instead of using decreased accuracy (DA) in the Eq. 2 of Supplemental Note, "positive" and "negative" are using decreased true positive rate (recall) and decreased true negative rate (specificity), respectively. MDA, the mean decrease of accuracy; MDGI, the mean decrease of Gini importance.

The importance of each feature for making correct classification was measured with MDA and MDGI (see Supplemental Note below). The larger of the value is, the more important the feature is in classifying protein pairs. From these results, we can see that IAS and PAS are the two most important features.

Supplemental Table S7. PPI prediction by overlap with SVM and RF.

Model	Precision	Accuracy
**SVM_{loc}**	0.947 (4226/4426)	0.919 (8749/9518)
**RF_{8loc}**	0.823 (2619/3181)	0.800 (5484/6854)
**SVM_{rand}**	0.794 (2667/3359)	0.708 (6735/9518)
**RF_{rand}**	0.940 (3078/3273)	0.920 (6309/6854)
**SVM_{loc} & RF_{8loc}**	0.980 (1857/1894)	0.676 (4634/6854)
**SVM_{rand} & RF_{rand}**	1.0 (1260/1260)	0.713 (4884/6854)

PPI detection performances by combining SVM and RF are examined on the two datasets, PPI_{loc} and PPI_{rand}. Precision is defined as the number of true interacting pairs predicted over the number of true interacting pairs and wrongly predicted interacting pairs (these numbers are shown in the parentheses of the precision column). Accuracy is defined as the number of corrected prediction (true positive and true negative) over the total number of data (including true positive, true negative, false positive, and false negative).

Supplementary Table S8 (in a separate Excel file) The BioGRID data sets used by support vector machine (SVM) and random forest (RF) with different sequence identify cutoffs.

Supplemental Figure S1. The distribution of the similarity of Cellular Component terms of predicted *Arabidopsis* interlogs.

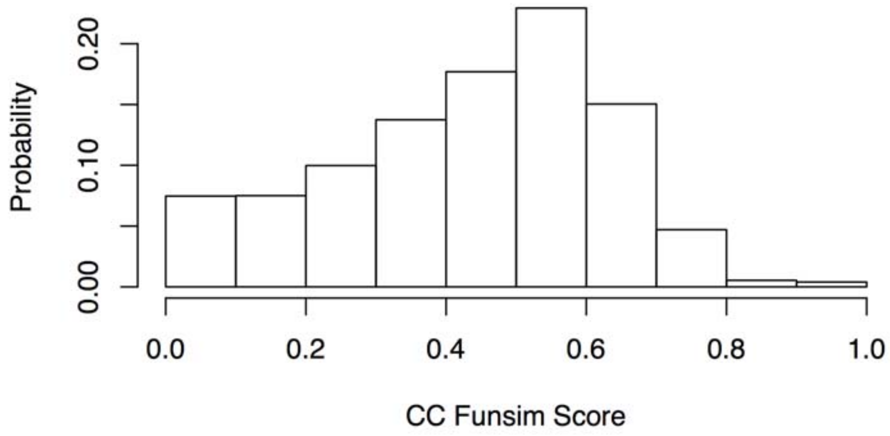

PPIs predicted in three papers, Geisler-Lee et al. [1], De Bodt et al. [2], Dutkowski et al [3] were analyzed. There were in total 50,949 PPIs predicted in the three papers, among which 2,786 pairs had CC GO term annotations. The FunSim score of only CC terms was computed. From this plot, we decided to use 0.4 as a cutoff to pre-screen protein pairs for the genome-scale screening of the three genomes, *Arabidopsis*, soybean, and corn. 61.31% of PPIs in the distribution had the score above 0.4.

Supplementary Table S9. The percentage of known PPIs among all possible protein pairs in well-studied organisms.

Organism	Number of Proteins	Number of PPIs	Percentage of experimentally identified PPIs over all possible protein pairs (%)
Arabidopsis thaliana	*27,636*	35,896	0.00939%
Homo sapiens	*20,213*	332,829	0.163%
Escherichia coli (K12)	*4,140*	12,801	0.149%
Saccharomyces cerevisiae (S288c)	*6,002*	108,088	0.600%
Drosophila melanogaster	*13,931*	47,068	0.0485%
Mus musculus	*22,089*	38,587	0.0158%

The number of proteins are taken from the KEGG database. The number of PPIs are taken from the BioGRID database.

Supplementary Table S10. The summary of predicted PPIs in *Arabidopis* with the interlog concept.

**Authors**	**Number of predicted PPIs**	**Reference organisms**
Geisler-Lee et al.	19,979	*S. cerevisiae, C. elegans, D. melanogaster, H. sapiens*
De Bodt et al.	18,674	*S. cerevisiae, C. elegans, D. melanogaster, H. sapiens*
Dutkowski et al.	14,009	*S. cerevisiae, C. elegans, D. melanogaster, H. sapiens, M. musculus, R. norvegicus*

Supplementary Table S11. Commonly predicted PPIs by using interlogs and PPIP.

**Method Combination**	**Number of commonly predicted PPIs**
Geisler-Lee & De Bodt	934
Geisler-Lee & Dutkowski	294
De Bodt & Dutkowski	188
PPIP & Geisler-Lee	118
PPIP & De Bodt	208
PPIP & Dutkowski	47

Supplementary Table S12, S13, S14 (in a separate Excel file). Predicted PPIs in *Arabidopsis thaliana* with different levels of confidence.

S12: PPI predictions with two additional evidence in *Arabidopsis thaliana*.

S13: PPI predictions with additional evidence in *Arabidopsis thaliana.*

S14: PPI predictions with no known evidence in *Arabidopsis thaliana.*

Supplementary Table S15, S16, S17 (in a separate Excel file). Predicted PPIs in *Zea mays* (corn) with different levels of confidence.

S15: PPI predictions with two additional evidence in *Zea mays* (corn).

S16: Predicted PPIs with additional evidence in *Zea mays* (corn).

S17: Predicted PPIs with no known evidence in *Zea mays* (corn).

Supplementary Table S18. Functional analysis of proteins in PPIs of corn and soy bean in Fig. 4.

Sub-networks	Number of proteins	Number of common GO terms	Number of common GO terms with P-value < 0.001	Function
**Corn 1**	101	5	5	flavonoid glucuronidation, flavonoid biosynthetic process, cellular glucuronidation, and quercertin 3-O-glucosyltransferase activity
**Corn 2**	59	8*	6*	RNA metabolic process and RNA secondary structure unwinding
**Corn 3**	41	2	2	proteolysis and protein catabolic process
**Corn 4**	23	92	21	MAPK signaling, protein phosphorylation, neuron development, pathway in response to stimuli
**Soybean 1**	145	87	87	MAPK signaling pathway in response to stimuli
**Soybean 2**	41	5	5	flavonoid glucuronidation, flavonoid biosynthetic process, cellular glucuronidation, and quercertin 3-O-glucosyltransferase activity
**Soybean 3**	20	17	12	RNA processing and intracellular protein transport
**Soybean 4**	9	9	9	signal transduction and cell communication in response to the stimulus

The “Number of common GO terms” column shows the predicted GO terms by PFP shared by all proteins involved in the subnetwork. For the corn subnetwork 2, there was no commonly shared GO term, but there are 8 commonly predicted biological process GO terms that are shared by 57 out of 59 proteins. The p-value indicates the rarity of the GO term in the protein set considering the number of proteins in the set, the number of proteins with that GO term in the organism, and the number of proteins in the organism (the function enrichment analysis).

Supplementary Table S19, S20, S21 (in a separate Excel file). Predicted PPIs in *Glycine max* (soybean) with different levels of confidence.

S19: PPI predictions with two additional evidence in *Glycine max* (soybean).

S20: Predicted PPIs with an additional evidence in *Glycine max* (soybean).

S21: Predicted PPIs with no other known evidence in *Glycine max* (soybean).

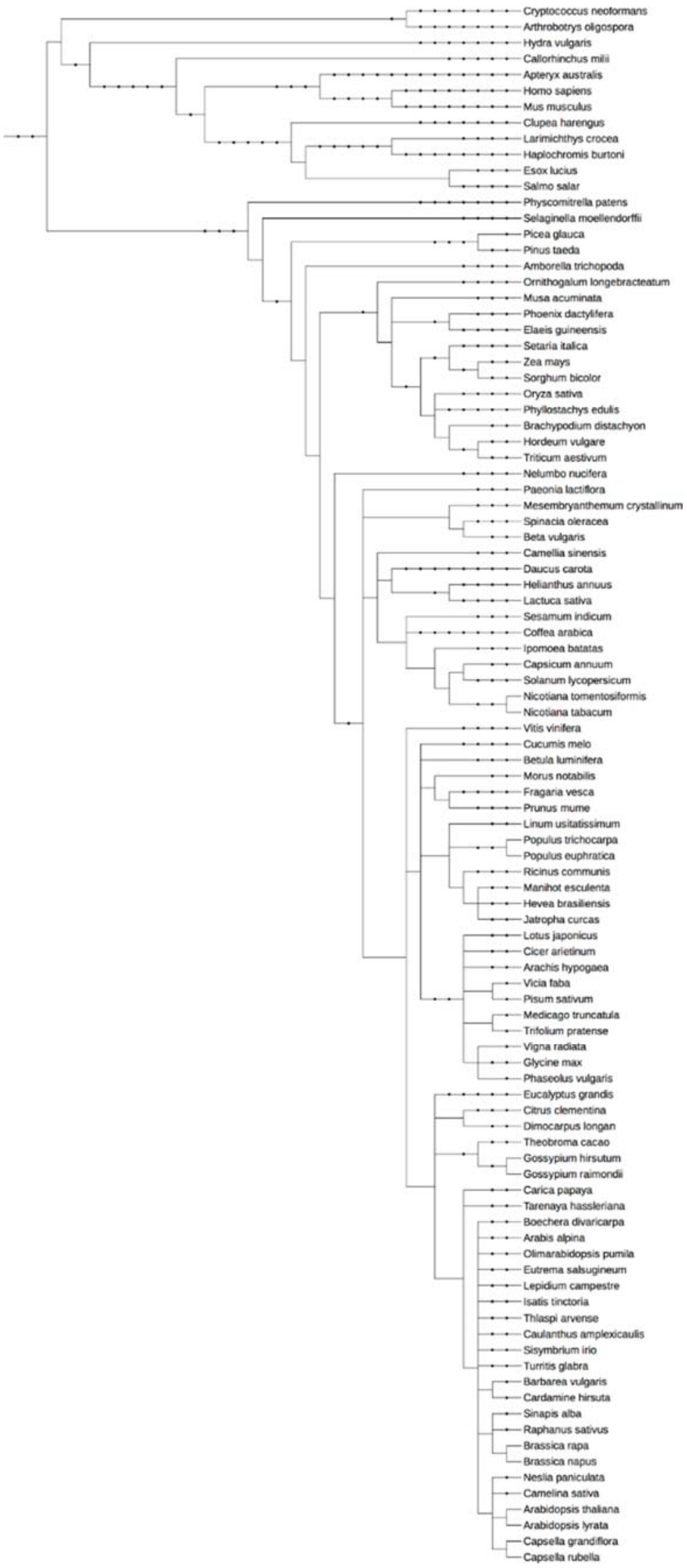

Supplementary Figure S2. The phylogenetic tree of selected 100 reference organisms used to compute phylogenetic profile of proteins. The tree was generated with phyloT (http://phylot.biobyte.de/).

Supplementary Note. The random Forest algorithm.

Random forest consists of many decision trees and takes a majority vote from decisions made by individual decision trees. There are three hyperparameters to decide: ntree, the number of decision trees used; mtry, is the number of features used in each decision tree; and cutoff, the fraction of the votes to make a prediction in the majority voting step. The figure below shows a schematic diagram of random forest.

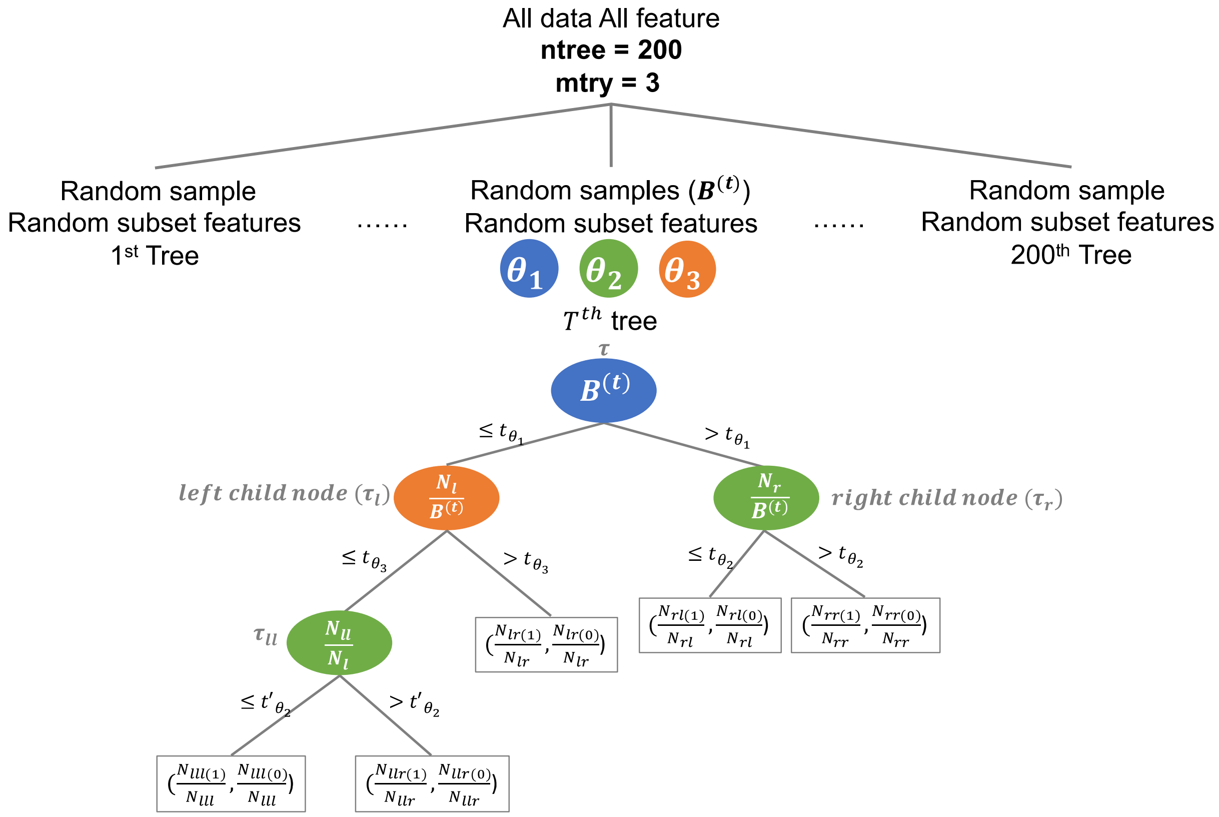

The schematic view of random forest algorithm. In this example, ntree = 200 and mtry = 3. N_l and N_r are the number of samples sending to the left child node (τ_l) and right child node (τ_r) in the splitting event at the node τ with cutoff value t_{θ_1}. Similarly, N_{ll} is the number of samples sending to the left child node (τ_{ll}) of the node τ_l at the splitting event with a cutoff value t_{θ_3}. $N_{lll(1)}$ and $N_{lll(0)}$ are the number of samples with label "1" and "0" after splitting at the node τ_{ll} with the cutoff value t'_{θ_2}.

Random forest can assess the importance of variables in making correct decisions using two metrics, mean decrease of accuracy (MDA) (Eq. 2) and mean decrease of Gini importance (MDGI) (Eq. 7) [4].

For the tth tree, the decrease of accuracy (DA) by permuting the feature x_j, which means shuffling the feature values with other data points, is defined as [5]:

$$DA^{(t)}(x_j) = \frac{\sum_{i \in B^{(t)}} I\left(y_i = \widehat{y_i}^{(t)}\right)}{B^{(t)}} - \frac{\sum_{i \in B^{(t)}} I(y_i = \widehat{y_{i,\pi_j}}^{(t)})}{B^{(t)}} \qquad (1)$$

where $B^{(t)}$ is the number of samples drawn randomly from the training data; y_i is the true label of the ith sample; $\widehat{y_i}^{(t)}$ is the predicted label of the ith sample before permuting the feature x_j; $\widehat{y_{i,\pi_j}}^{(t)}$ is the predicted label of the ith sample after permuting the feature x_j. This equation calculates how much the accuracy decreased among $B^{(t)}$ samples for the tth tree. Therefore, the mean decrease of accuracy is defined as:

$$MDA\ (x_j) = \frac{\sum_t^{ntree} DA^{(t)}(x_j)}{\widehat{\sigma_{DA}}\sqrt{ntree}} \qquad (2)$$

where $\widehat{\sigma_{DA}}$ is the estimated standard deviation of $DA^{(t)}(x_j)$. A large MDA indicates that the feature is important for classifying the dataset.

The second metric, MDGI, is computed from the Gini impurity of each node of trees in random forest. The Gini impurity at a node τ of the tth decision tree is computed as:

$$i(\tau) = 1 - \left(\frac{N_1}{N}\right)^2 - \left(\frac{N_0}{N}\right)^2 \qquad (3)$$

where N_1 and N_0 are the number of samples with label “1” and “0” at the τ node, respectively, and N is the total number of samples at the τ node. Thus, it measures how impure the data, i.e. samples with different labels are at the node. Using Eq. (3), the impurity decrease $\Delta i(\tau)$ is defined as the decrease of the impurity by splitting and sending the samples to two children nodes τ_l and τ_r using a threshold t_θ for a feature x_j:

$$\Delta i(\tau) = i(\tau) - \left(\frac{N_l}{N}\right) i(\tau_l) - \left(\frac{N_r}{N}\right) i(\tau_r) \qquad (4)$$

In order to obtain the maximum Δi, i.e. to identify the most effective feature and the threshold value for splitting the dataset, all combinations of the feature chosen from “mtry” features at the node τ and its threshold t_θ are tested. The decrease in Gini impurity resulting from the optimal split $\Delta i_{x_j}(\tau, t)$ is accumulated for all nodes τ from the tth decision tree, and accumulated for all the trees in the forest. The accumulated quantity is called the

Gini Importance (I_G):

$$I_G(x_j) = \frac{1}{N_t} \sum_t \sum_{\tau \in t} \Delta i_{x_j}(\tau, t) \quad (5)$$

Using Eq. 5, the degrease of Gini importance (DGI) is computed as the difference from the Gini importance when the values of the variable $x_j$are permuted:

$$DGI^{(t)}(x_j) = I_{G,x_j}^{(t)} - I_{G,\pi_j}^{(t)} \quad (6)$$

where $I_G^{(t)}$ is the Gini importance of jth feature with samples from t^{th} tree, and $I_{G,\pi_j}^{(t)}$ is the Gini importance of the jth permutated feature. Then, the mean decrease of Gini importance (MDGI) is calculated as the sum of DGI normalized by the number of trees ($ntree$) and the standard deviation of DGI ($\widehat{\sigma_{DGI}}$) (Eq. 5).

$$MDGI(x_j) = \frac{\sum_t^{ntree} DGI^{(t)}(x_j)}{\widehat{\sigma_{DGI}} \sqrt{ntree}} \quad (7)$$

A large MDGI indicates that the feature is informative for making correct classification.

References:

1 Geisler-Lee, J. *et al.* A predicted interactome for Arabidopsis. *Plant Physiology* **145**, 317-329 (2007).

2 De Bodt, S., Proost, S., Vandepoele, K., Rouzé, P. & Van de Peer, Y. Predicting protein-protein interactions in Arabidopsis thaliana through integration of orthology, gene ontology and co-expression. *BMC genomics* **10**, 288 (2009).

3 Dutkowski, J. & Tiuryn, J. Phylogeny-guided interaction mapping in seven eukaryotes. *BMC bioinformatics* **10**, 393 (2009).

4 Breiman, L. Random forests. *Machine learning* **45**, 5-32 (2001).

5 Strobl, C., Boulesteix, A.-L., Kneib, T., Augustin, T. & Zeileis, A. Conditional variable importance for random forests. *BMC bioinformatics* **9**, 307 (2008).
